# Studies in Blood Pressure, Physiological and Clinical

**Published:** 1916-12

**Authors:** 


**Studies in Blood Pressure, Physiological and Clinical.** By George Oliver, M.D. Lond., F.R.C.P. Edited by W. D. Halliburton, M.D., F.R.S. Third Edition. London: H. K. Lewis & Co. Ltd. 1916. Demy 8vo, pp. xxiii. 240. Price 7s. 6d. net.

The untimely death of the author left the major part of this volume ready for the printer. The new edition was placed in the hands of Prof. W. D. Halliburton, who has added certain chapters to complete the views of the author. The obituary notice from *The Lancet*, January 8th, 1916, and an article of the same date on "The Measurement of Blood Pressure," sums up much of Dr. Oliver's work. After thirty-three years of strenuous life at Harrogate he retired to London and Sidmouth for the winter months, and died at Farnham, Surrey, on December 27th, 1915. He wrote much on the Harrogate waters, and a book on the practice of bedside urine testing by means of Oliver's testpapers reached a fourth edition. But it was always the blood and its circulation that most attracted his attention, and various instruments, now well known and in common use, were brought under medical notice as clinical instruments. Of these the sphygomanometer is by far the most important, and will long be associated with his name; but the fallacies in its use are many, and experience alone can enable the observer to make full use of the phonendoscope attachment to the mercurial manometer.

Another instrument, the arteriometer, is devised for the purpose of determining the internal diameter or calibre of the artery. Chapter x. describes its construction and use, and the author believed that the conjoint use of this apparatus with the manometer would be a source of sustained interest in enabling one to follow the relation between the vaso-motor state of the arteries and the blood-pressure, and thus daily exemplify in practice our physiological knowledge of that relation. Chapter xi., on venous and capillary pressure, shows that the author was a keen observer of nature, and did not always depend on any elaborate mechanical apparatus for his results.

